# Snow Metrics as Proxy to Assess Sarcoptic Mange in Wild Boar: Preliminary Results in Aosta Valley (Italy)

**DOI:** 10.3390/life13040987

**Published:** 2023-04-11

**Authors:** Annalisa Viani, Tommaso Orusa, Enrico Borgogno-Mondino, Riccardo Orusa

**Affiliations:** 1 Institute for Piedmont, Liguria, Aosta Valley (IZS PLV) S.C Valle d’Aosta—CeRMAS (National Reference Center for Wildlife Diseases), Località Amerique 7/G, 11020 Quart, Italy; riccardo.orusa@izsto.it; 2 GEO4Agri DISAFA Laboratory, Department of Agricultural, Forest and Food Sciences (DISAFA), University of Turin, Largo Paolo Braccini 2, 10095 Grugliasco, Italy; tommaso.orusa@gmail.com (T.O.); enrico.borgogno@unito.it (E.B.-M.)

**Keywords:** wild boar, parasitology, *Sarcoptes scabiei*, GIS and Remote Sensing, snow metrics, Theia CNES CesBIO, Orfeo ToolBox

## Abstract

The widespread diffusion of the wild boar on the Italian territory and its consistent use for hunting have created the possibility to conduct multiple studies on the pathologies afflicting this ungulate. Nevertheless, in the last two decades, only some pathologies such as classical and African Swine Fever, Tuberculosis, Brucellosis from *Brucella suis* have benefited from substantial public funding and the consequent great interest from the scientific world, while less attention was addressed to parasitic diseases including sarcoptic mange. Therefore, to fill this gap, the purpose of this study was to contribute to the knowledge of sarcoptic mange in the wild boar population in Aosta Valley in the Northwest of Italy, including sympatric species as foxes. Due to past field surveys, it has been possible to find a possible role of snow metrics in the spread of this pathogen. Even if there are only empirical evidence and the mechanism remain unknown remote sensing analysis considering snow metrics were performed to provide to veterinarians, foresters, biologists, and ecologists new tools to better understand wield board dynamics and join to ordinary tool an instrument to enhance management and planning strategies. The snow metrics (SM) were derived from USGS NASA Landsat 8 L2A retrieved from Theia CNES platform and processed in Orfeo Toolbox LIS extension package. The relationship between SM and the disease spread was tested per each Aosta Valley municipality obtaining LISA maps for each hunting season. The results have showed that this parasite is present in an endemic form even if with rather low prevalence values, equal to 1.2% in the season hunting season 2013/2014, and equal to 7.5% in the hunting season 2014/2015. Moreover, within simultaneous given values of SM, sarcoptic mange seem to find good conditions for spreading.

## 1. Introduction

Domestic and wild animals are affected by different types of mange. Six types are reported in the literature: sarcoptic mange, demodectic mange, chorioptic mange, psoroptic mange, notoedric mange and otodectic mange. In this work we focused on the study of sarcoptic mange within the wild boar population of the Valle d’Aosta, also carrying out collateral biomolecular investigations on mites collected in one other sympatric specie: the fox.

Sarcoptic mange is caused by the mite *Sarcoptes scabiei* and has been found in 10 orders, 27 families and 104 species of domestic and wild animals [1,2,3,4,5,6,7,8,9,10,11].

If some isolated cases of mange have been reported in different hosts, in others the parasitosis can acquire an epizootic course. These animals include the coyote (*Canis latrans*), red fox (*Vulpes vulpes*), and wolf (*Canis lupus*) in North America [12,13,14,15], the artic fox (*Alopex lagopus*) [16], the red fox and the wolf in Europe [17,18], the red fox and the dingo (*Canis familiaris dingo*) in Australia [19], the lynx (*Lynx lynx*) in Europe [20,21], lion (*Panthera leo*) and cheetah (*Acinonyx jubatus*) in Africa [22], Alpine chamois (*Rupicapra rupicapra*), Alpine ibex (*Capra ibex*) and Iberian ibex (*Capra pyrenaica*) in Europe [23,24], wild boar (*Sus scrofa*) in Europe [24], wombat (*Wombatus ursinus*) and koala (*Phascolarctos cinereus*) in Australia [25], the mountain gorilla (*Gorilla gorilla berengei*) and the chimpanzee (*Pan* spp.) in Africa [26], and the impala (*Aepyceros melampus*), the alcephalus (*Alcelaphus buselaphus*), the “springbok” or springbok (*Antidorcas marsupialis*), the wildebeest (*Connochaetes taurinus*), the black buffalo (*Syncerus caffer*), the eland antelope (*Taurotragus oryx*), the greater kudu (*Tragelaphus strepsiceros*), Thompson’s and Grant’s gazelle (*Gazella thompsoni* and *Gazella gazella*, respectively) and sable antelope (*Hippotragus niger*) in Africa [27].

Sarcoptic mange can cause high mortality in animals such as red fox (*Vulpes vulpes*), Alpine chamois (*Rupicapra r. rupicapra*) and Iberian ibex (*Capra pyrenaiaca*) [28]. In wild boar, however, parasitosis is not associated with high mortality [29] rates despite high morbidity [30]. Although several cases involving wild 42 have been reported in the literature [31,32] it is possible to state that sarcoptic mange has so far been poorly described in this species [33].

It is estimated that around 300 million people in the world are affected by this pathology, and that its presence is linked to conditions of poverty and overcrowding, rather than to poor hygienic conditions [33]. This parasitosis has been known in mankind since ancient times. Thanks to archaeological studies of Ancient Egypt, it is thought to have been discovered more than 2500 years ago. The first literary reference to scabies appears in the Bible (Leviticus, 3rd book of Moses) ca. 1200 BC Among the ancient authors who have described its manifestations, we find the Arab doctor Abu Hasan el Tabar around 970 BC, and a few years later Hildegard of Bingen and the Moro doctor Ibn Zuhr. Later, the Greek philosopher Aristotle (384–322 BC) will speak of “mites” which “escape from small pimples if they are stung”. The attribution of the name “scabies” is due to the Roman doctor Celsus, who described its characteristics. In 1687, Bonomo and Cestoni, two naturalists of the time, described the etiology of scabies in a letter, an unofficial document. Only in 1868, two centuries later, did the physicist and dermatologist Ferdinand Karl Franz Schwarzmann Ritter von Hebra publish a medical treatise, in which he precisely illustrated the biological cycle and the epidemiology of the parasite [34].

During mange infestations, guests become infected by direct contact with sick subjects, presumably following the transfer of the larval forms found on the skin surface. Nonetheless, cases of passive transfer have been observed, as the mite is able to survive in the environment for short periods. Under controlled environmental conditions, in the presence of an optimal microclimate, these mites can survive in the environment for a few weeks [35].

Unfortunately, little is known about interspecific transmission from domestic animals to wild animals, while more information is available regarding transmission between wild animals themselves.

Regarding the first case, some wild ungulates, including European mouflon (*Ovis musimon*), red deer (*Cervus elaphus*) and fallow deer (*Dama dama*) were affected by an epizootic, subsequently attributed to domestic goats infested by the mite [36]. In this regard, an attempt to experimentally infest a group of chamois (*Rupicapra rupicapra*) with *Sarcoptes scabiei* deriving from naturally infested domestic goats has been described in the literature. The opposite route of transmission, chamois vs. goat, on the other hand, had already been demonstrated by another experimental study [24]. To corroborate the hypothesis of passage from wild to domestic, the literature reports an episode of sarcoptic mange in northern Italy which affected domestic goats, and which was attributed to their contact with wild animals [37].

The infestation of wild animals, unlike that of the domestic one, is characterized by a high mortality and greater morbidity of the affected subjects, which is essentially due to the difficulty of carrying out therapeutic interventions to limit the acariasis. Furthermore, we must not forget that, in nature, a co-infection between *S. scabiei* and other types of ecto-endo-parasites is not uncommon. Despite this, the exact type of interaction that occurs between the different parasites is not known, due to the lack of studies on the subject [38]. In addition, the presence of mites can cause direct or indirect damage, such as irritation, skin inflammation, itching, self-trauma, nervousness, and allergic responses [39].

The presence of *S. scabiei* in the ecosystem generates complex “Parasite Navigating Webs” (P-NW). An appropriate knowledge of the P-NW is essential to understand the dynamics of the parasite in the environment, in particular its ways and conditions of transmission be-tween animals of the same or different species, to be able to control the disease. Based on the definition of occurrence and recurrence of a disease as described [39,40], the manifestation of a case of sarcoptic mange can be the result of the outbreak of an “outbreak” within a human or animal population, or simply be the result of a sneaky infection hitherto remained silent for the most varied reasons. *Sarcoptes* re-emergence is defined as the re-emergence of this mite after a decline in incidence [41]. In ruminants, the possibility that these parasites, adapted to a main host, can infest other sympatric species has been demonstrated in various “outbreaks” occurring in Europe [36,42], and supported by the results obtained following experimental infestations [24]. Human migrations themselves may be at the root of the outbreak of new cases of scabies. The epidemic can then also be an apparent appearance or reappearance of the infestation, where the *Sarcoptes* infestation was already present, and its new appearance can be due to a modification of the previous balance established between host and parasite, rather than to a decrease in the host’s immune defenses, or to the selection of a more pathogenic and drug-resistant variant of the parasite [13,43].

*Sarcoptes scabiei* is taxonomically divided into different varieties based on the host origin, although from the morphological point of view these mites do not present evident differences. The characterization of the mitochondrial DNA haplotypes and the study of the microsatellite allele frequency allowed to demonstrate a significant association between *S. scabiei* and the host species or the geographic locations.

The wild boar population present in Valle d’Aosta probably derives from subjects irradiated from Savoy as per the first report by Fosson which took place in 1995 of wild boars present in Aosta Valley already in 1931 in Fenis (AO), and in more recent times also from bordering Piedmont. Currently the wild boar is present in a stable form throughout the region, from the valley floor up to altitudes exceeding 2000 m. As elsewhere, the main objective of wild boar management in Aosta Valley is the numerical containment of the species, especially in agricultural areas and in the most valuable faunal environments (Regional Wildlife and Hunting Plan, five-year period 2008–2012).

**Figure 1:**
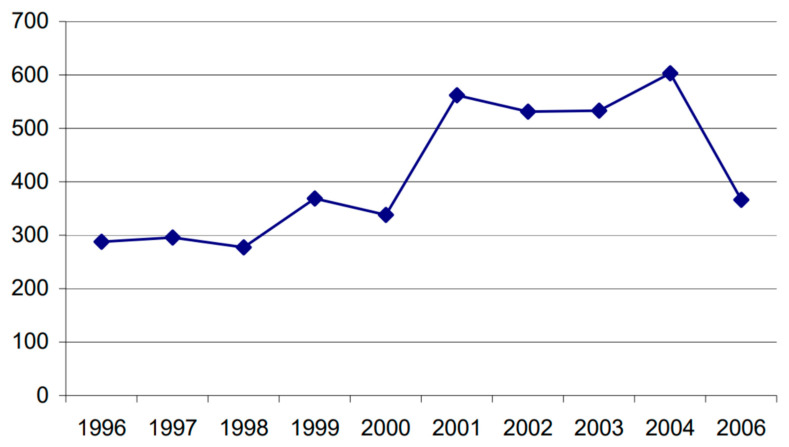
Wild boars surveyed with the counting of footprints in the snow in the Aosta Valley. Little ones are excluded (Regional Wildlife and Hunting Plan, five-year period 2008–2012).

The numbers of wild boar present in Aosta Valley have been estimated since 1996 by the Forestry Corps of Aosta Valley, through the survey of tracks on the snow in winter. The results of the latest official estimates published are shown in Figure 1. The data must be under-stood as the certain minimum number of animals present, which obviously represents an underestimation of the total. In the data available, infants (subjects in the first semester of life) represent on average 10% of the total number of individuals, juveniles (subjects in the second semester of life) 25%, sub-adults (subjects in the second year of life) 32% and adults 29%. The in determinate make up 4–5% of the sample.

In Aosta Valley, wild boar hunting is allowed in two ways: stalking (or wandering), generally open from mid-September to mid-November, based on a specific selective sampling plan, and driven hunting, usually allowed from mid-November until the end of January within specific sectors and carried out by teams designated by the Regional Committee for hunting management. The species is also subject to control activities, carried out outside the hunting season by the Forestry Corps of the Aosta Valley.

For better management of the ungulates and in consideration of the distribution of the protected territory, among other things, wildlife control centers have been created in which animals killed during the hunting season are conferred for the collection of more information and samples. These Centers have been established in the municipalities of Morgex, Aymavilles, Etroubles, Valpelline, Nus, Châtillon, Challand-Saint-Victor, Pont-Saint-Martin, and Gaby. The control centers of Etroubles, Aymavilles and Pont-Saint-Martin are supervised by the CeRMAS (National Reference Center for Wildlife Diseases) veterinarians of the Experimental Zooprophylactic Institute of Piedmont, Liguria, and Valle d’Aosta for the implementation of regional health monitoring.

It’s worth to note that the bloomed of sciences and technologies applied to GIS and Remote Sensing has opened new frontiers in the eco-epidemiological sector, by providing powerful new tools for zoonoses risk spreading and animal disease surveillance. Geospatial analysis techniques and the use of satellites provide useful methods for collecting and manage the information necessary for epidemiological studies. Still little explored in the veterinary field is the use and development of applications and methodologies based on Earth Observation Data (EO data). In this regard, within the scope of the European space program “Copernicus” and other historical missions such as NASA USGS Landsat that provide medium-high geometric resolution geospatial data make it possible to exploit and expand the ordinary techniques of risk analysis translating them into a technology transfer for the veterinarian sector. To date, in fact, only a few applications have been explored with the use of medium-low resolution data in the context of NASA Terra and Aqua MODIS missions. Although promising, the MODIS data are not very suitable for analysis and development of models at a local scale due to their geometric resolution, especially at the Alpine municipal level. Therefore, in order to avoid bias related to poor geometric resolution it is necessary to use higher resolution data in the Alpine area for analyzes on a local scale [44,45].

The snow cover is an important driver of many eco-pathological, climatic and hydrological processes in mountain regions and in high latitude areas. Snow is a critical component of the climate system, provides fresh water for millions of people globally, and affects forest and wildlife ecology. Snowy regions are typically data sparse, especially in mountain environments. Remote-sensed snow cover data are available globally but are challenging to convert into accessible, actionable information. Nowadays, the huge development of cloud computing and developing in highly performance algorithm to detect snow changes permits to extract snow metrics in order to perform eco-pathological studies. One of the most important variables from snow satellite observations are the snow metrics such as: (a) the snow cover duration map (SCD), that is to say the overall number of days with a continuous presence of snow on the ground, (b) the date of snow disappearance snow melt-out date (SMD), defined as the last date of the longest snow period, (c) the date of snow appearance snow onset date (SOD), defined as the first date of the longest snow period.

The aim of this work was to depict the prevalence of *Sarcoptes scabiei* in the wild boar population in the Aosta Valley, also analyzing the infestation risk factors (sex, age, month of culling, weather conditions). Furthermore, molecular investigations were carried out on mites collected from infested wild boars and foxes, to evaluate whether they belonged to a single parasitic population or to differentiated populations, and therefore contribute to the understanding of the origin of sarcoptic mange in the Aosta Valley wild boar population. To make this comparison, we performed the molecular characterization of mites (*S. scabiei*) using nine microsatellite markers, as described by [38]. Additionally, a geospatial analysis concerning on snow metrics (SM) and the detection of the disease for each municipality and through hunting seasons were carried out. In particular, a relationship was investigated by developing LISA maps.

## 2. Materials and Methods

### 2.1. Samples Collection and Research of Mites

#### 2.1.1. Wild Boar

Samplings were carried out for the research of *S. scabiei* in wild boars. The study involved the collection of an auricle from each wild boar hunted during the 2013/2014 and 2014/2015 hunting seasons, for a total of 205 pavilions. The samples were taken when the animals were presented at the Hunted Game Control Centre, located in Pont-Saint-Martin (AO), annexed to the headquarters of the local Forestry Station. The sampling took place under the supervision of an official veterinarian working at the Centre, belonging to the IZS CeRMAS.

Further samples (always pinnae) were recovered at the “La Kiuva” game meat processing center, located in Arnad (AO), with the prior agreement and authorization of the owner of the facility and the AUSL (local health unit). The collected auricles, suitably identified, were stored, and kept in a freezer (−20 °C) at the CeRMAS laboratories and finally transported to the Parasitic Dis-eases laboratory of the Department of Veterinary Sciences, University of Turin, for carrying out the analyses. Here the samples were thawed and subjected to skin scraping on the inner surface of the auricle with the aid of a scalpel. 

The scraped material was introduced, together with the scalpel blade, into 12 cc plastic test tubes containing 5–6 cc of 10% NaOH solution, subsequently incubated in a thermostat until all the inserted material was completely digested (3 h at 37 °C or 12 h at 22 °C).

At the end of the digestion, the samples were centrifuged at 3000 rpm for 15 min. Finally, the supernatant was eliminated, the sediment mixed and, using a Pasteur pipette, two-three drops of the same were deposited on a slide and covered with a coverslip to be examined under an optical microscope at 40X. Four slides were examined for each sample.

**Figure 2:**
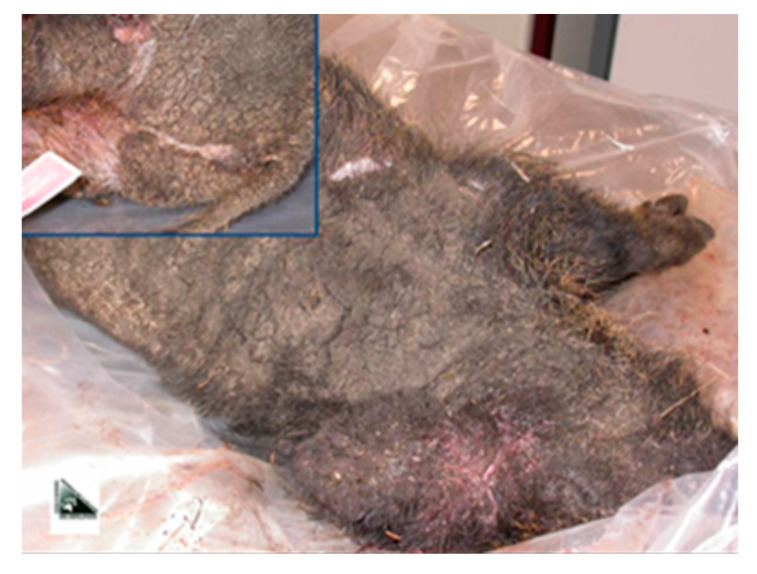
Macroscopic pictures of sarcoptic mange dermatitis (*Sarcoptes scabiei*) at various stages of severity in wild boar.

A further contribution to the study was provided by CeRMAS, with the provision of two whole carcasses of wild boars (sex: male; age class: “red” (subjects of 6–12 month); municipalities of origin: Donnas and Perloz) subjected to euthanasia by Forestry Corps agents, upon notification by citizens. Both subjects presented generalized lesions, compatible with a suspicious pattern of sarcoptic mange (Figure 2). The carcasses were delivered, also in this case, to the Parasitic Diseases laboratory of the Department of Veterinary Sciences of Grugliasco for diagnosis confirmation and further specialist investigations to be carried out.

Skin scrapings were performed at the said laboratory on the inner face of the auricle and at the level of the hyperkeratotic lesions present on the back, sides, withers, tarsi, and tail. Subsequently, skin scrapings were managed and analyzed as described above for pinnae.

#### 2.1.2. Fox

**Figure 3:**
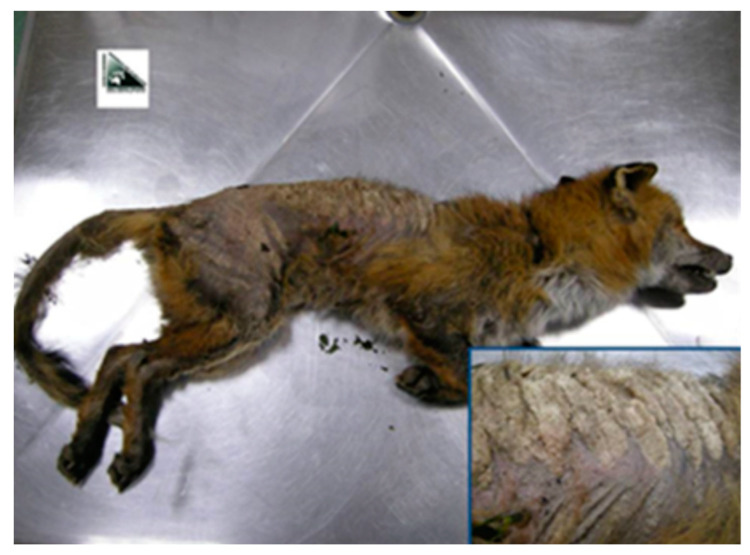
Macroscopic pictures of sarcoptic mange dermatitis (*Sarcoptes scabiei*) at various stages of severity in fox.

CeRMAS has made directly available to the Parasitic Diseases laboratory of the Department of Veterinary Sciences, University of Turin, skin samples previously isolated from 9 foxes found dead in the Valle d’Aosta area and preserved by freezing. In all cases they were animals with full-blown scabies, debilitated and heavily infested (Figure 3). Confirmatory skin scrapings were performed as described for the previous boars.

### 2.2. Biomolecular Investigations

Samples positives for *S. scabiei* (12 wild boars, 9 foxes) were subjected to molecular biology investigations.

First, single mites (at least 10 per sample) were collected under the stereoscopic microscope and then stored in 70% ethanol inside the Eppendorf. The extraction of DNA from single mites for the execution of a multiplex PCR was performed using the HotSHOT Plus Thermal SHOCK technique [38].

Each mite isolated from the skin was transferred to a 0.5 mL tube. Following the addition of 25 µL of lysis buffer (25 mM NaOH/0.2 mM EDTA pH 12) three cycles of thermal shock were carried out. Each of these cycles consisted of a first very rapid freezing “step” (−80 °C for 2 min) and a subsequent very rapid thawing “step” (+95 °C). Subsequently the samples were incubated for 45 min at 95 °C, cooled for 5 min at 4 °C and the pH was neutralized with 25 µL of a second buffer (40 mM Tris HCl, pH 5). The DNA thus obtained was stored at −20 °C, until its use for multiplex PCR (see below). The molecular characterization of *S. scabiei* mites was performed using nine mite-specific molecular markers (microsatellites), as described by [38].

Microsatellites were amplified by multiplex PCR, using the following protocol:

Buffer (10×)
Final concentration: 1×
dNTP (10 mM): 0.2 mM
Primer F (5 μM): da 0.15 a 0.30 μM
Primer R (5 μM): da 0.15 a 0.30 μM
TAQ (5 U/μL): 0.5 U/reaction
Mite’s DNA: 20 n/g

It was chosen to use a reaction volume of 15 µL.

The cycling conditions were as follows:

95 °C × 15 min

94 °C × 30 s

55 °C × 45 s → 37 cycles

72 °C × 1.5 min

72 °C × 7 min

**Figure 4:**
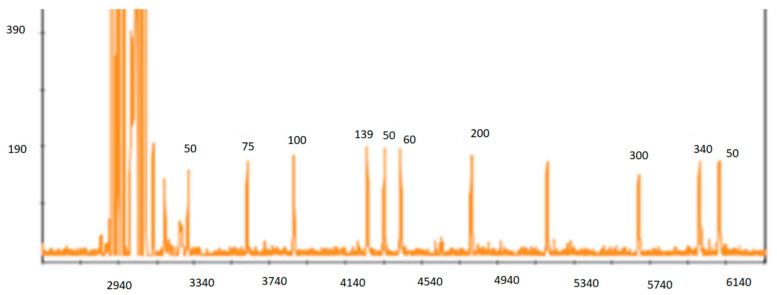
Internal marker representation or size standard.

Before submitting the amplicons to the automatic analyzer (sequencer), it was necessary to carry out a dilution of the sample with a mixture of deionized formamide (Hi Di formamide, Applied Biosystems, Foster City, CA, USA) and an internal marker or size standard (Figure 4).

Formamide has the function of maintaining the amplicon in a state of denaturation (monofilaments) during the subsequent phases. The internal marker is used as a reference to be able to assign sizes to the fragments.

**Figure 5:**
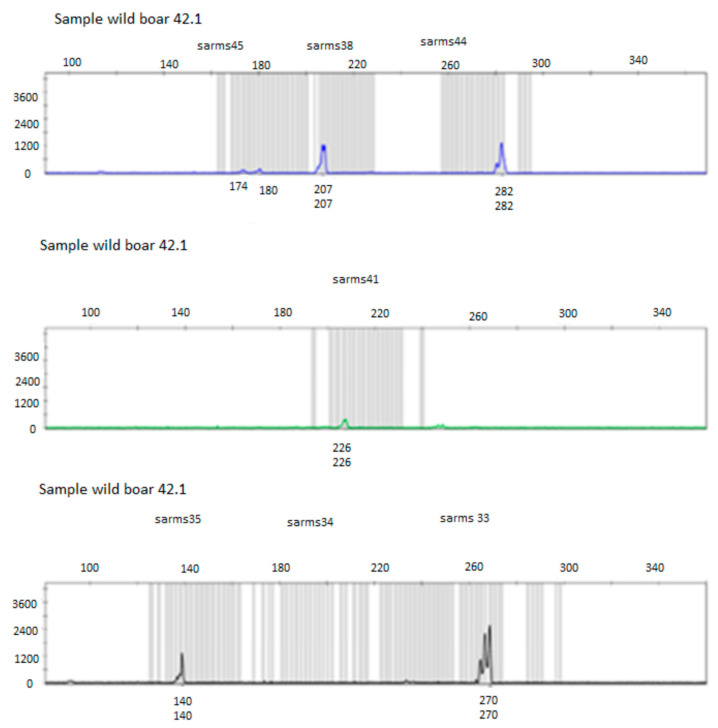
Electropherogram of the 9 microsatellites for the mite n° 1 related to the wild boar 42.1.

The electropherogram obtained for each sample was analyzed with the GeneScan^®^ Analysis software 3.1.2 (Applied Biosystems, Foster City, CA, USA) which allows to associate a size to each peak based on the internal marker (Figure 5).

Identification of alleles for each locus was performed semi-automatically using Genotyper 3.7 software (Applied Biosystems, Foster City, CA, USA); this program identifies alleles and creates a typing table. The possibility of setting objective criteria for the recognition of the peaks as alleles allows the reduction of errors due to the variability in the reading criteria adopted by different operators or by the same operator at different times. In particular, the binning analysis allowed to examine the microsatellite profiles for each marker with fluorochrome. Binning is the one-dimensional grouping, based on size, of fragments compiled from multiple samples. The analysis of the bins obtained allows to generate a list of alleles representative of the population under study. When bin analysis is conducted, the system groups fragments according to their size, to form bins characterized by the average size and a standard deviation of the fragments included in the bins. The analysis of each allelic peak and its dimensioning in base pairs allowed to obtain the genotype for each mite.

Frequencies of each allele were calculated using MSA software v. 3.12 (Dieringer 2003 Microsatellite analyzer (MSA): a platform independent analysis tool for large microsatellite datasets).

The genetic distance between individuals was calculated based on the distances DAS, Dc, Dm and Ds [43] evaluated with the software POPULA-TIONS v. 1.2.32 (http://www.bioinformatics.org/project/?group_id=84, last accessed on 27 March 2023). From the distance matrices a phylogenetic tree was constructed using the Neighbor-joining method. The tree was visualized with the MEGA v 4.0 program (Philadelphia, PA, USA). Furthermore, the STRUCTURE v. 2.3.3 (Santa Clara, CA, USA), which allows to obtain simulations about the possible number of populations into which the dataset of analyzed samples could be divided.

### 2.3. Geospatial Analysis

#### 2.3.1. Study Area

**Figure 6:**
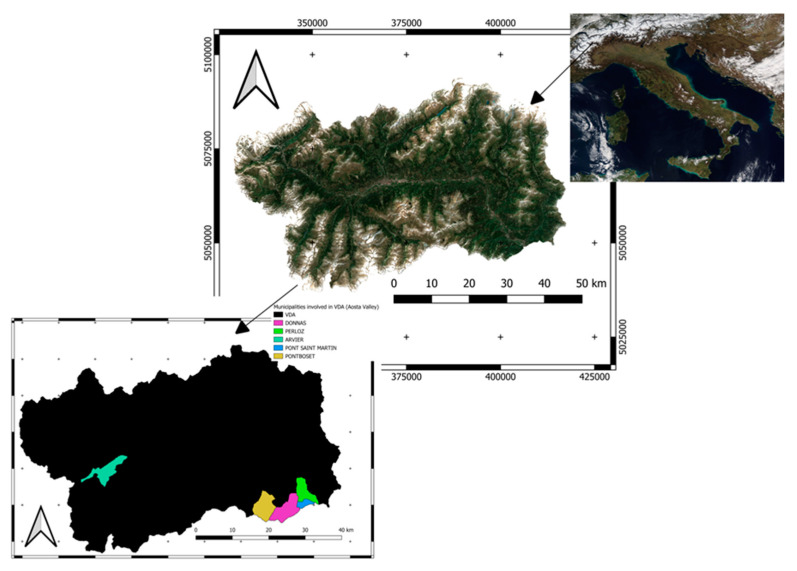
The study area involves the Aosta Valley autonomous region in NW Italy (ED50 UTM 32N) with particular regard on the municipality of Donnas, Perloz, Arvier, Pontboset, Pont-Saint-Martin EPSG:23032.

Geospatial analysis has involved the Aosta Valley autonomous region in NW Italy, the smallest Italian region in terms of surface extent, located in the mid-west of the Alps. It is surrounded by the four highest mountain massifs in Italy: Mont Blanc, which is also the highest peak in Europe, the Cervino-Matterhorn (4478 m), Monte Rosa (4634 m) and Gran Paradiso (4061 m) [44,45,46]. The conformation of the entire regional territory is the result of the work of many glaciations. The data were collected in the following municipalities and on which this work has been focused: Donnas, Perloz, Arvier, Pontboset, Pont -Saint-Martin retrieved from the SCT regional Geoportal (https://geoportale.regione.vda.it/, last accessed on 30 January 2023). Here below it has been reported the study area (see Figure 6).

#### 2.3.2. Earth Observation data Snow Metrics

Geospatial analyzes were carried out by adopting Earth Observation data from the USGS NASA’s mission Landsat 8 OLI. In particular, the input files were Landsat-8 level-2A products downloaded from the CNES Theia Land Data Centre (https://theia.cnes.fr/atdistrib/rocket/#/home, last accessed on 30 January 2023) and the Digital Surface Model (DSM) retrieved from the Aosta Valley SCT Geoportal (https://geoportale.regione.vda.it/, last accessed on 30 January 2023) resampled at 30 m (staring from a native resolution product of 2 m) [44,45,46,47]. Landsat 8 data ranging from the hunting seasons 2013–2014 and 2014–2015 respectively, were temporally aggregated and stacked in order to compute Snow Metrics (here in after called SM) thanks to the Orfeo Toolbox (OTB) remote module developed by CESBIO and CNES called Let-It-Snow (LIS). This plugin implementable in OTB version ≥7.4 permits to have an operational snow cover detection from optical multispectral level 2A products and derive snow product such as: snow cover (SC) and/or snow fractional snow cover (FSC). Moreover, snow metrics such as: the snow cover duration map (SCD), pixel values within 0-number of days corresponding the number of snow days. The date of snow disappearance Snow Melt-Out Date (SMD), defined as the last date of the longest snow period. The dates are given in number of days since the first day of the synthesis. the date of snow appearance Snow Onset Date (SOD), defined as the first date of the longest snow period. The dates are given in number of days since the first day of the synthesis. And finally, the number of clear observations (NOB) to compute the SCD, SMOD and SOD syntheses. The algorithm works with multispectral remotely sensed images, which include at least a channel in the visible part of the spectrum and a channel near 1.5 µm (referred to as shortwave-infrared or SWIR). It takes the following as input: a L2A product, including the cloud and cloud shadow mask (referred to as an “L2A cloud mask” in the following), the green, red and SWIR bands from the flat-surface reflectance product. These images are corrected for atmospheric and terrain slope effects [46]. The slope correction is important in mountain regions since it enables to use the same detection thresholds whatever the sun-slope geometry. The red and green bands are resampled with the cubic method to a pixel size of 20 m by 20 m to match the resolution of the SWIR band. A digital elevation model (DEM) and other optional parameters to improve the snow detection over different biophysical surfaces. It is worth noting that, FSC were computed per each hunting season stack of Theia product Landsat 8 OLI Level-2A. To do this in Let-It-Snow algorithm the following optional parameters were included: the tree cover density (TCD) retrieved from Land Copernicus platform (https://land.copernicus.eu/, last accessed on 30 January 2023), the water mask retrieved from Aosta Valley Land cover [47], and the relief shadow mask obtained from the Aosta Valley DSM. The output generated was two FSC stacks (Level-2B snow product). These Level-2B FSC stacks were then adopted to get SM. The SM syntheses were temporally aggregated (level-3A products) derived from individual SM stacked after gap filling. The three final outputs were: SOD, SMD, SCD.

The snow detection is based on the Normalized Difference Snow Index (NDSI, Donmez) and the reflectance in the red band. The NDSI is defined as:

$$\mathrm{NDSI}=\frac{\rho_{\mathrm{green}}-\rho_{\mathrm{SWIR}}}{\rho_{\mathrm{green}}+\rho_{\mathrm{SWIR}}}$$

where $\rho_{\mathrm{green}}$ (or ρ_SWIR_) is the slope-corrected surface reflectance in the green band (or SWIR at 1.6 µm). The NDSI expresses the fact that only snow surfaces are very bright in the visible and very dark in the shortwave infrared. Turbid water surfaces like some lakes or rivers may also have a high NDSI value; hence an additional criterion on the red reflectance is used to avoid false snow detection in these areas. A cloud-free pixel is classified as snow if the following condition is true:

$$\left(\mathrm{NDSI}>n_{i}\right){\;\mathrm{AND}\;\rho }_{\mathrm{red}}>{\;r}_{i}$$

where n_i_ and r_i_ are two parameter pairs (minimum value of snow reflectance per each band in the stack). Otherwise, the pixel is marked as “no snow”. The parameter values were set based on previous studies with Landsat [48] and by visually checking many snow maps and snow fraction histograms. Finally, the algorithm includes some additional steps to recover those pixels from the L2A cloud mask and reclassify them as snow or no snow. This step is important because it substantially increases the number of observations. A pixel from the L2A cloud mask cannot be reclassified as snow or no snow if any of these conditions are satisfied: coded as “cloud shadow” in the L2A cloud mask; coded as “high-altitude cloud” (or “cirrus”) in the L2A cloud mask; not a “dark cloud”.

The cloud shadows are excluded because the signal-to-noise ratio can be very low in these areas. The high clouds are excluded because they can have a similar spectral signature as the snow cover, such as a high reflectance in the visible and a low reflectance in the SWIR. This type of cloud is detected Landsat-8 L2A products based on the spectral band centered on the 1.38 µm wavelength. In order to analyze a possible relationship between SM and the presence of sarcoptic mange for each hunting season, zonal statics have been performed considering each municipality. The municipal-based and non-punctual analysis were dictated by the fact that the samples have not been pointly georeferenced. Nevertheless, it is worth noting that wild boar normally lived in a buffer zone not far from they have been hunted or die as suggested by a well know ecological and ethological scientific literature concerning on this type of wildlife [49]. In order to test the existence of a spatial correlation, LISA (Local Indicators of Spatial Autocorrelation) maps at the municipal scale computed in QGIS v. 3.16 [50] were created per each hunting season, LISA varies between −1.0 and +1.0 and its numerator is interpreted as the covariance between contiguous units.

As follow:

$$\mathrm{LISA}=\frac{N}{\sum_{i}\sum_{j}W_{\mathrm{ij}}}\;\frac{\sum_{i}\sum_{j}W_{\mathrm{ij}}\left(x_{i}-\mu \right)\left(x_{i}-\mu \right)}{\sum_{i}{\left(x_{i}-\mu \right)}^{2}}$$

where:

N: is the number of geographic units;

xi: is the variable that describes the phenomenon under study in region i;

μ: represents the sample mean and therefore (xi − μ) is the deviation from the mean of the variable of interest;

W_ij_ is the weight matrix which in many cases is equivalent to a binary matrix i,j where weights inversely proportional to the distance between the point i and point j (where i is different from j).

## 3. Results

Hunting season 2013/2014

Out of a total of 83 samples collected, *n* = 1 auricle was positive for the presence of *Sarcoptes scabiei* Therefore, the prevalence of *S. scabiei* infestation in wild boars hunted during the 2013/2014 hunting season was 1.2% (1/83). The wild boar positive for *S. scabiei* was male and belonging to the “red” age group (6–12 months). 

Hunting season 2014/2015

Out of a total of 120 samples collected, *n* = 8 auricles were positives in the presence of only *Sarcoptes scabiei*. Furthermore, *n* = 1 auricle was positive for the presence of both mites (*Sarcoptes scabiei* and *Demodex pylloides*). Therefore, the prevalence of *S. scabiei* infestation in wild boar hunted during the 2014/2015 hunting season was 7.5% (9/120). The wild boars positive for *S. scabiei* are 5 females and 4 males; 7 (77.7%) of these animals belong to the “red” age group, one is a “sub-adult” (12–36 months) and one an “adult 1” (>36 months).

Statistical analysis of prevalence data (Fisher Exact Test one and two tails) highlighted the following:

(1) the prevalence for *S. scabiei* varied (*p*-value = 0.023 and *p*-value = 0.029) in the two hunting seasons. It should be noted, however, that in the first hunting season the “reds” were underrepresent-ed.
(2) the prevalence for *S. scabiei* did not vary in the two sexes (*p*-value = 0.550 and *p*-value = 1.000);
(3) the prevalence for *S. scabiei* varied in relation to age, with a significantly higher risk in “reds” than in subadults (*p*-value = 0.005 and *p*-value = 0.009; RR = 10.46) and in adults (*p*-value = 0.002 and *p*-value = 0.002; RR = 12.15).

### 3.1. Biomolecular Investigation

The raw data obtained from the analysis for 9 microsatellites of a total of 33 mites (*Sarcoptes scabiei*), of which 12 from wild boar and 21 from fox, are reported in Table 1.

In the mite dataset analyzed, the number of alleles detected by the 9 MS markers ranged from 2 to 5, with Sarms 35, 36 and 44 recognized as the least polymorphic and Sarms 33 as the most polymorphic (see Table 2 and Table 3).

**Figure 7:**
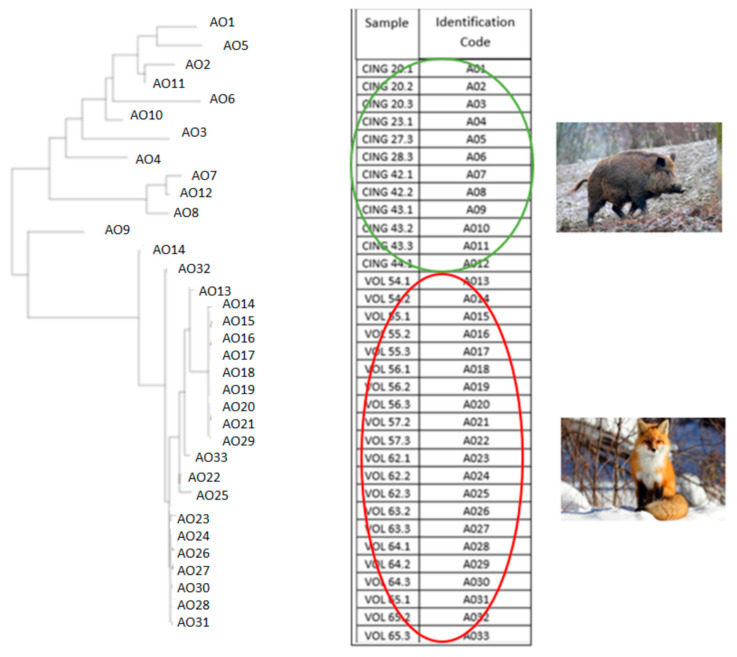
Phylogenetic tree of 33 specimens of *Sarcoptes scabiei* isolated from 7 wild boars and 8 foxes, with relative legend of the simplified codes of each sample for data entry into the software used (CING = wild boar, VOL = fox).

**Figure 8:**
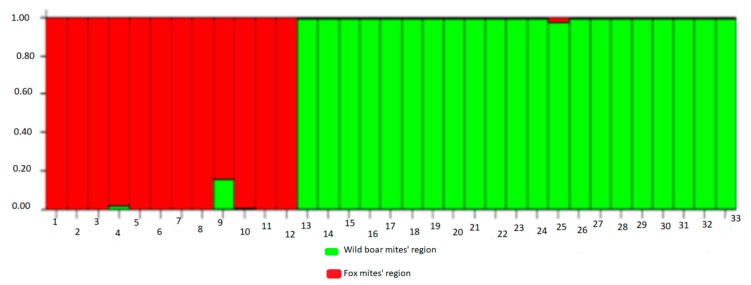
Subdivision of the mites (*Sarcoptes scabiei*) analyzed in populations, as returned by the STRUCTURE software.

Starting from all the mites analyzed, a phylogenetic tree was obtained (Figure 7), and the subdivision into populations as returned by the STRUCTURE version 2.3.3 software, which highlights two distinct populations of mites, one of which originates from wild boars and the other than foxes (Figure 8).

**Figure 9:**
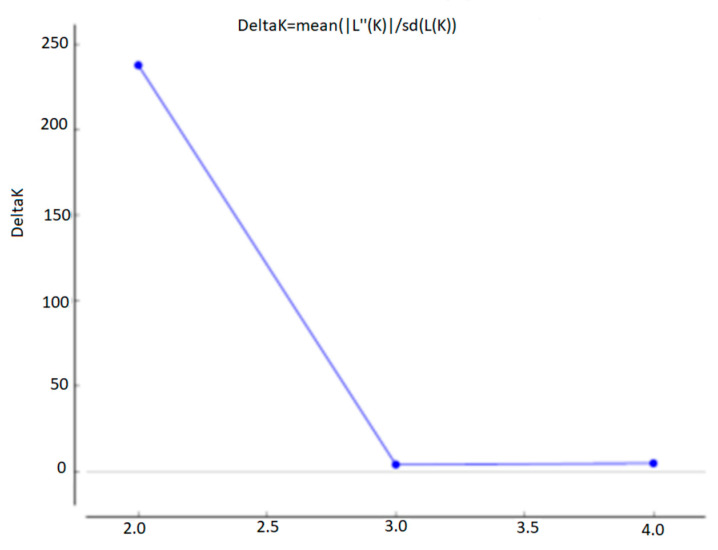
Graphic indicating the most probable number of populations (k) according to Evanno’s method (https://taylor0.biology.ucla.edu/struct_harvest/, last accessed on 26 March 2023).

The subdivision into clusters produced by the distance analysis is supported by the simulations obtained with STRUCTURE, according to which 2 host-specific populations can be distinctly identified in the sample (Figure 9).

### 3.2. Geostatistic Results

**Figure 10:**
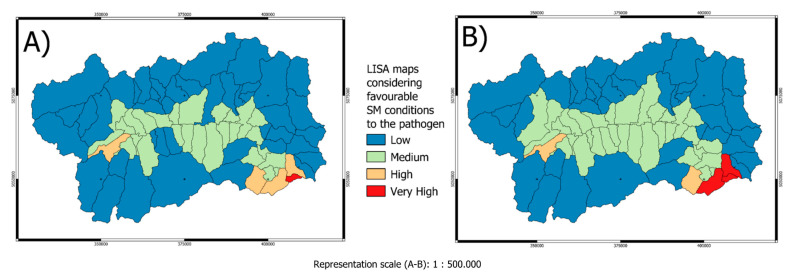
LISA maps in the municipalities of Aosta Valley considering the relationship between snow metrics within given ranges and the pathogen (ED50 UTM 32N) (**A**) hunting season 2013–2014 (**B**) hunting season 2014–2015 EPSG:23032.

Geostatistical analysis showed that in the years in which wild boar are found positive and outbreaks are particularly strong in each municipality, the following conditions occur simultaneously 10 > SCD > 110 75 > SOD > 110 140 > SMD < 240. In fact, by performing a linear regression considering the location and these variables for the years studied an R^2^ = 0.71 and 0.69 have been found. This observation repeats itself spatially and temporally in all the municipality considered within the study area. This condition seems to constitute an important parameter for risk and potential transmissibility analysis of the pathogen. Therefore, to demonstrate a possible relationship between certain SM values mapped by remote sensing and presence of the pathogen associated with considerable virulence LISA was calculated to test the existence of a spatial correlation for all hunting season considered. It is worth noting that in all cases LISA > 0.90, therefore statistically significant. Here below (see Figure 10) it has been reported the LISA maps. It is interesting to note that in the municipality where the above-mentioned conditioned was observed a high presence of the pathogen was detected. Furthermore, future studies would be highly recommended in order to better understand the possible link between SM and the pathogen presence.

## 4. Discussion

This work contributes to the knowledge of sarcoptic mange in the wild boar population of the Valle d’Aosta.

The results show how this parasitosis is present in an endemic form even if with rather low prevalence values, equal to 1.2% in the 2013/2014 hunting season, and equal to 7.5% in the 2014/2015 hunting season. Low prevalence values of sarcoptic mange infestation were also re-ported by [51] who, between 1994 and 1998 in Poland, conducted a study to investigate the prevalence of parasites such as *Dermacentor reticulatus*, *Demodex phylloides*, *Ixodes ricinus* and *S. scabiei* in free-living wild boars. It emerged that *S. scabiei* appeared sporadically in these animals, as did *D. reticulatus*, *D. phylloides* and *I. ricinus*, on the other hand, presented prevalence values of 30% and 17%, respectively.

The Aosta Valley wild boars affected by this infestation mostly belong to the “red” age group (second semester of life), which testifies that the young age of the wild boars represents a risk factor, probably due to a certain degree of immaturity of the immune system. In experimental models, a good functionality of the immune system is considered essential to effectively counteract the multiplication of parasitic mites [35]. Poorly is known concerning on free-living wild boars, but in these animals living in captivity were observed that sarcoptic mange occurs mainly in a subclinical form, often going unnoticed and that the clinical signs of parasitosis are more evident in immunocompromised subjects (malnourished and/or with forms of endoparasitosis, during the lactation period) [29,33].

On the other hand, the sex of the animals does not seem to play a determining role in the pathogenesis of the infestation in Aosta Valley wild boars, since both male and female wild boars are equally affected. In the literature, a greater sensitivity to infections has often been found in male animals, in which there are more evident and serious clinical symptoms than in female subjects [52]. In general, androgens tend to make the immune response less efficient [53]; moreover, maintaining a greater body mass requires male animals to make a greater energy contribution, which, added to the cost of the immune response, exposes them to a greater risk of depletion of body reserves [54].

The geographical distribution of the wild boar on the territory of the Autonomous Region of Valle d’Aosta appears very wide (Regional Wildlife and Hunting Plan, 2008–2012): these animals form a single population within which the sarcoptic mange can now be considered endemic.

As for the origin of the sarcoptic mange among the wild boars of the Aosta Valley, two hypotheses could be formulated. The first is that the source of the infestation was traced back to infested foxes. In fact, parasitosis is well documented in this species also at a regional level [55]. Diseased foxes have often been considered the primary source of sarcoptic mange infestation in other animals, including wild carnivores, dogs, and variable hares (*Lepus timidus*) [16,18,21]. Furthermore, recent genetic studies on mite populations have suggested that cross-transmission be-tween sympatric host species is possible within some trophic chains [41,56]. Evidence of a “prey-predator” transmission has been recently provided by [57]. The fox is not a usual prey of the wild boar, but the latter is also a necrophagous species, especially during the winter, when the mortality rate due to sarcoptic mange in the various affected species tends to grow up [28,33,41]. For this reason, a direct contact between wild boar and dead foxes, with inter-transmission of the mite, is at least plausible.

The second hypothesis (the most credible a priori) would instead believe that wild boars became infested following contact with carrier or sick animals belonging to the same species, possibly coming from territories bordering the Autonomous Region of Valle d’Aosta. Indeed, cases of sarcoptic mange have been documented in wild boars in Switzerland [32], France [30], Piedmont and Liguria [30,58]. In this regard, starting from 2012 a survey like the present work was conducted in Liguria and specifically in the province of Imperia. This survey envisaged active surveillance during three consecutive hunting seasons (between 2012 and 2014) by specially trained hunters, with a definitive diagnosis performed at the IZS of Imperia, in collaboration with the University of Turin, starting from a sample of skin with the presence of lesions. 14 “clinical” cases of mange were reported, both sarcoptic (*n* = 6) and demodectic (*n* = 8) which came from all the municipalities subjected to surveillance and affected 0.7% of the animals collected (N = 2009). Passive surveillance in the same areas allowed us to highlight two more cases of mange of clinical severity (one case of demodectic mange and one of sarcoptic mange).

The molecular investigations we performed on specimens of *S. scabiei* collected on wild boars and foxes from the regional territory seem to confirm the second hypothesis, as the mites tested unequivocally belong to two populations that are well differentiated in relation to the host species. Only one mite from wild boar A09 was “midway” between the mite population of wild boars and the mite population of foxes. Only five out of nine microsatellites were compared for this specific sample, because 4 were not suitable for evaluation.

The existence of two different mite populations in wild boars and foxes is further evidence of the species-specificity of these parasites towards the parasitized hosts, as always reported in the bibliography. Having said this, the possibility of evolution and change of this “rule” must be highlighted, as has already occurred in other European and non-European contexts (see above), and it is therefore appropriate that in conditions of sympatry between guests, both infested with sarcoptic mange, the epidemiological studies are completed with insights of a molecular nature (molecular epidemiology).

Snow metrics, and in particular the presence of certain conditions regarding the timing of the appearance, disappearance and persistence of the snowpack seem to play a role in the distribution of the pathogen affecting the ecology of the wild boar. However, it should be noted that the data available are fewer and not enough to provide strong statistical evidence. Therefore, the results obtained must be considered as preliminary and deserve more in-depth studies and insights by the scientific community in order to achieve solid evidence on samples obtained over several years and in larger areas confirming or confuting the geospatially based results here obtained. Having said this, the use of remote sensing and GIS tools in the veterinary and eco-epidemiological fields can certainly constitute an added value to ordinary diagnostic techniques [59,60,61,62,63,64,65]. The technological transfer offered by spatial analyzes in terms of repercussions on the veterinary sector as highlighted in this study can certainly be positive. Therefore, further studies on this matter can certainly represent a key for a better understanding of the spread of the pathogen not only at local scale, but also at national and international level [66], allowing veterinary medicine and biology to interpenetrate new professionalisms and collaborations with other figures, also favoring an interdisciplinary approach necessary for understand complex systems and at the same time ensure innovation by guaranteeing a consistent and real One Health.

## 5. Conclusions

In conclusion, sarcoptic mange is endemic in Aosta Valley region (NW Italy) even if with rather low prevalence values during the hunting seasons considered. In particular, the prevalence of *Sarcoptes scabiei* is equal to 1.2% in the hunting season 2013/2014, and equal to 7.5% in 2014/2015.

Geospatially based analyses concerning on SM and their temporal and spatial simultaneously recurrence within certain given range regarding snow onset, melting and duration seem to play a role in the distribution of the pathogen affecting the ecology of the wild boar. Nevertheless, the suggested approach, deserves further and due more investigations in reason of the fact that the analyzed population is contained in only given municipalities and the number of animals found positive does not have a sample abundance to represent a statistically representative sample. 

## Figures and Tables

**Table 1:** Allele sizes of the 33 mites (*Sarcoptes scabiei*) examined (n = negative, WB = Wild Boar, F = Fox).

Sample	Host	ms33		ms35		ms36		ms37		ms38		ms40		ms41		ms44		ms45	
20.1	WB	274	274	140	140	275	275	174	174	207	207	237	237	238	238	282	282	174	184
20.2	WB	234	274	140	140	275	275	174	174	207	207	215	237	238	238	282	282	184	184
20.3	WB	234	274	140	148	275	275	174	174	209	209	215	237	238	238	282	282	184	184
23.1	WB	232	272	140	148	273	273	170	174	209	211	237	237	236	238	282	282	184	184
27.3	WB	n	n	n	n	n	n	174	174	207	207	237	237	240	240	n	n	n	n
28.3	WB	n	n	140	140	273	273	172	172	207	207	n	n	238	238	n	n	184	184
42.1	WB	270	270	140	140	273	273	174	174	207	207	215	221	226	226	282	282	174	180
42.2	WB	270	270	140	140	273	273	174	174	207	211	215	221	226	226	282	282	174	184
43.1	WB	232	274	n	n	n	n	170	170	207	207	n	n	n	n	262	282	184	184
43.2	WB	232	274	n	n	n	n	170	174	207	207	n	n	n	n	282	282	184	184
43.3	WB	232	274	140	140	n	n	174	174	207	207	215	237	n	n	282	282	184	184
44.1	WB	270	270	140	140	273	273	174	174	207	207	215	215	226	226	282	282	174	180
54.1	F	232	232	148	148	283	283	170	170	170	n	n	215	215	234	234	n	n	186
54.2	F	232	232	n	n	281	281	170	170	170	211	211	215	215	234	234	n	n	n
55.1	F	232	232	148	148	281	281	170	170	170	211	211	215	215	234	234	262	262	186
55.2	F	232	232	148	148	281	281	170	170	170	211	211	215	215	234	234	262	262	186
55.3	F	232	232	148	148	281	281	170	170	170	211	211	215	215	234	234	262	262	186
56.1	F	232	232	148	148	281	281	170	170	170	211	211	215	215	234	234	262	262	186
56.2	F	232	232	148	148	281	281	170	170	170	211	211	215	215	234	234	262	262	186
56.3	F	232	232	148	148	281	281	170	170	170	211	211	215	215	234	234	262	262	186
57.2	F	232	232	148	148	281	281	170	170	170	211	211	215	215	234	234	262	262	186
57.3	F	n	n	148	148	n	n	170	170	170	211	211	215	215	234	234	n	n	n
62.1	F	232	232	148	148	283	283	170	170	170	211	211	215	215	234	234	262	262	186
62.2	F	232	232	148	148	283	283	170	170	170	211	211	215	215	234	234	262	262	186
62.3	F	232	232	n	n	283	285	170	170	170	211	211	215	215	234	234	262	262	186
63.2	F	232	232	148	148	283	283	170	170	170	211	211	215	215	234	234	262	262	186
63.3	F	232	232	148	148	283	283	170	170	170	211	211	215	215	234	234	262	262	186
64.1	F	232	232	n	n	283	283	170	170	170	211	211	215	215	234	234	262	262	186
64.2	F	232	232	148	148	281	281	170	170	170	211	211	215	215	234	234	262	262	186
64.3	F	232	232	148	148	283	283	170	170	170	211	211	215	215	234	234	262	262	186
65.1	F	232	232	148	148	283	283	170	170	170	211	211	215	215	234	234	262	262	186
65.2	F	232	232	n	n	283	283	170	170	170	211	211	215	215	234	234	262	262	n
65.3	F	232	232	148	148	281	283	170	170	170	211	211	215	215	234	234	262	262	186

**Table 2:** Alleles found for the 9 microsatellite markers and their frequency starting from the analysis of 12 specimens of *Sarcoptes scabiei* isolated from 7 wild boars (n = negative).

Locus	Allele	Frequency
Sarms 33	274	0.292
	234	0.083
	232	0.167
	272	0.042
	270	0.250
	n	0.167
Sarms 35	140	0.667
	148	0.083
	n	0.250
Sarms 36	275	0.250
	273	0.417
	n	0.333
Srms 37	174	0.750
	170	0.167
	172	0.083
Sarms 38	207	0.792
	209	0.125
	211	0.083
Sarms 40	237	0.375
	215	0.292
	221	0.083
	n	0.250
Sarms 41	238	0.375
	236	0.042
	240	0.083
	226	0.250
	n	0.250
Sarms 44	282	0.792
	262	0.042
	n	0.167
Sarms 45	174	0.167
	184	0.667
	180	0.083
	n	0.083

**Table 3:** Alleles found for the 9 microsatellite markers and their frequency starting from the analysis of 21 *Sarcoptes scabiei* isolated from 8 foxes (n = negative).

Locus	Allele	Frequency
Sarms 33	232	0.952
	n	0.048
Sarms 35	148	0.810
	n	0.190
Sarms 36	283	0.476
	281	0.405
	285	0.024
	n	0.095
Sarms 37	170	1.000
Sarms 38	211	0.952
	n	0.048
Sarms 40	215	1.000
Sarms 41	234	1.000
Sarms 44	262	0.857
	n	0.143
Sarms 45	186	0.857

## Data Availability

Not applicable.
